# Antipsychotic Prescriptions for Prenatal and Postpartum Women in Japan: A Large Claims Database Study

**DOI:** 10.1002/prp2.70336

**Published:** 2026-10-28

**Authors:** Ryo Obara, Takamasa Sakai, Tomofumi Ishikawa, Kei Morishita, Motohiko Adomi, Azusa Hara, Saya Kikuchi, Natsuko Kobayashi, Noriyuki Iwama, Genki Shinoda, Aoi Noda, Masatsugu Orui, Mami Ishikuro, Hiroaki Tomita, Nariyasu Mano, Shinichi Kuriyama, Taku Obara

**Affiliations:** ^1^ Division of Molecular Epidemiology Graduate School of Medicine, Tohoku University Sendai Miyagi Japan; ^2^ Drug Informatics, Faculty of Pharmacy Meijo University Nagoya Aichi Japan; ^3^ Laboratory of Biomolecule and Pathophysiological Chemistry, Graduate School of Pharmaceutical Sciences Tohoku University Sendai Miyagi Japan; ^4^ Department of Pharmaceutical Sciences Tohoku University Hospital Sendai Miyagi Japan; ^5^ Department of Epidemiology Harvard T.H. Chan School of Public Health Boston Massachusetts USA; ^6^ Laboratory of Social Pharmacy and Epidemiology Showa Pharmaceutical University Machida Tokyo Japan; ^7^ Department of Psychiatry Tohoku University Hospital Sendai Miyagi Japan; ^8^ Department of Psychiatry Graduate School of Medicine, Tohoku University Sendai Miyagi Japan; ^9^ Department of Preventive Medicine and Epidemiology, Tohoku Medical Megabank Organization Tohoku University Sendai Miyagi Japan; ^10^ Center for Maternal and Perinatal Medicine Tohoku University Hospital Sendai Miyagi Japan; ^11^ Department of Disaster Public Health, International Research Institute of Disaster Science Tohoku University Sendai Miyagi Japan; ^12^ Department of Disaster Psychiatry, International Research Institute of Disaster Science Tohoku University Sendai Miyagi Japan

**Keywords:** antipsychotic, Japan, postpartum, prenatal, prescription, psychiatric disorders, schizophrenia

## Abstract

This study aimed to evaluate the prevalence of antipsychotic prescriptions before, during, and after pregnancy in Japan, with a focus on new prescriptions and continuation after pregnancy confirmation. We conducted a claims‐based analysis using a Japanese claims database (analytic period: 2010–2022). Antipsychotic prescription prevalence and new prescriptions were assessed from 180 days before pregnancy through 180 days postpartum. To assess continuation after pregnancy confirmation, prescription status was evaluated across gestational weeks 0–4, 5–13, and from 14 weeks to delivery. Among 120 735 women, 1698 (140.6/10 000 deliveries) received at least one antipsychotic prescription, including 809 (67.0/10 000 deliveries) during pregnancy. Prescription prevalence decreased during the first and second trimesters but increased postpartum. New prescriptions were less frequent during pregnancy but increased after delivery. Among 611 women prescribed antipsychotics during 0–4 weeks' gestation, 482 (78.9%) continued and 129 (21.1%) discontinued prescription during weeks 5–13; of those who continued prescription during 5–13 weeks, 334 (54.7%) continued until delivery. Evaluation of major indications showed that continuation until delivery was highest in those with schizophrenia (63.6%), followed by bipolar disorder (51.0%). This study provides the first comprehensive description of perinatal antipsychotic prescription trends in Japan. Substantial prescription discontinuation occurred after pregnancy confirmation. Increases in postpartum prescriptions might reflect new prescriptions or resumption among women who had discontinued treatment during pregnancy. These findings offer foundational epidemiological insights into real‐world perinatal antipsychotic use.

**Abbreviations**
DPA: dopamine partial agonist
ICD‐10: international classification of diseases, 10th revision
LAI: long‐acting injectable
MARTA: multi‐acting receptor‐targeted antipsychotic
SDA: serotonin–dopamine antagonist

## 1 Purpose

Depression, bipolar disorder, schizophrenia, and other psychiatric disorders are known to affect maternal self‐care, obstetric complications, child development, and childcare environment in the perinatal period [1, 2]. Untreated or insufficiently treated psychiatric conditions during pregnancy may lead to worsening of symptoms and adverse perinatal outcomes, making timely, appropriate treatment critical [3]. Pregnant individuals may consider discontinuing psychotropic medications, as well as other medications, because of concerns about their effects on fetal health [4]; they may also not take medications in the postpartum period due to concerns about breast milk transfer, or may discontinue breastfeeding. There is a higher incidence of antipsychotic discontinuation in pregnant versus non‐pregnant women [5]. Relapse and hospitalization following antipsychotic discontinuation during pregnancy have also been reported [6, 7]. The postpartum period is also associated with increased risk of relapse, first‐onset psychosis, and bipolar disorder, further highlighting the importance of careful pharmacological management during this period.

The risks and benefits of antipsychotic use during the perinatal period for mothers and infants should be considered. In Japan, the Perinatal Mental Health Consensus Guide 2023 [8] and the Clinical Guide for Women with Mental Health Problems during the Perinatal Period [9], along with other relevant guidelines [10, 11], state that many antipsychotics are generally considered appropriate to continue during pregnancy and lactation. However, despite these recommendations, reports on perinatal psychiatric drug prescriptions in Japan have examined only antidepressants and antiepileptics [4, 12, 13], and comprehensive data on antipsychotics remain scarce. Although some reports have examined antipsychotic use, detailed information on individual agents and their perinatal continuation, discontinuation, and resumption remains limited [14, 15, 16], making it difficult to assess real‐world prescription patterns during pregnancy.

Studies on antipsychotics prescribed to prenatal and postpartum women could provide important indicators regarding which are most commonly prescribed before, during, and after pregnancy, and catalyze programs to optimize antenatal prescriptions, including in Japan. Health administrative databases are important sources of data for the pharmacoepidemiological evaluation of drug exposure during pregnancy [17]. This study aimed to clarify perinatal antipsychotic prescription trends in women using a large administrative database, examining new prescriptions, continuation, discontinuation, and resumption after pregnancy confirmation.

## 2 Methods

Data were extracted from a large database of health insurance claims developed and maintained by JMDC Inc. in Tokyo, Japan [18]. This database includes all inpatient, outpatient, and pharmacy claims received from insurers and is one of the largest claims databases in Japan. Claims included diagnoses classified according to the International Classification of Diseases, 10th revision (ICD‐10), surgical and medical procedures, and prescribed medications. Standardized disease classification and anonymous record linkages were used [18]. More details regarding this database can be found in our previous reports [13, 19]. The Institutional Review Board of Tohoku University School of Medicine approved this study on January 22, 2024 (registration number: 2023‐1‐808), waiving the requirement for informed consent.

The dataset available on August 10, 2022, was used, which included 13 470 546 men and women covered under health insurance between January 2005 and February 2022 (Figure 1). Mothers whose children were born and could be identified were included; mothers can be linked to offspring in the JMDC claims database if their infants are enrolled with the same health insurer, allowing the identification of the month and year of birth (the date of birth is not available to avoid re‐identification). Women who met the following eligibility criteria were included: those whose infants were enrolled with the same health insurer during their birth month, whose dates of pregnancy onset and delivery could be estimated, whose first pregnancy between 2010 and 2022 was included (the number of women who gave birth before 2009 was limited), and who were members of one of several health insurance schemes exclusively from 180 days before pregnancy onset through 180 days postpartum. As no dates of pregnancy onset or delivery were available in the Japanese claims database, they were estimated using algorithms described elsewhere [12, 13, 19, 20] as detailed in the Appendix (Figure S1) [11, 21, 22].

**FIGURE 1:**
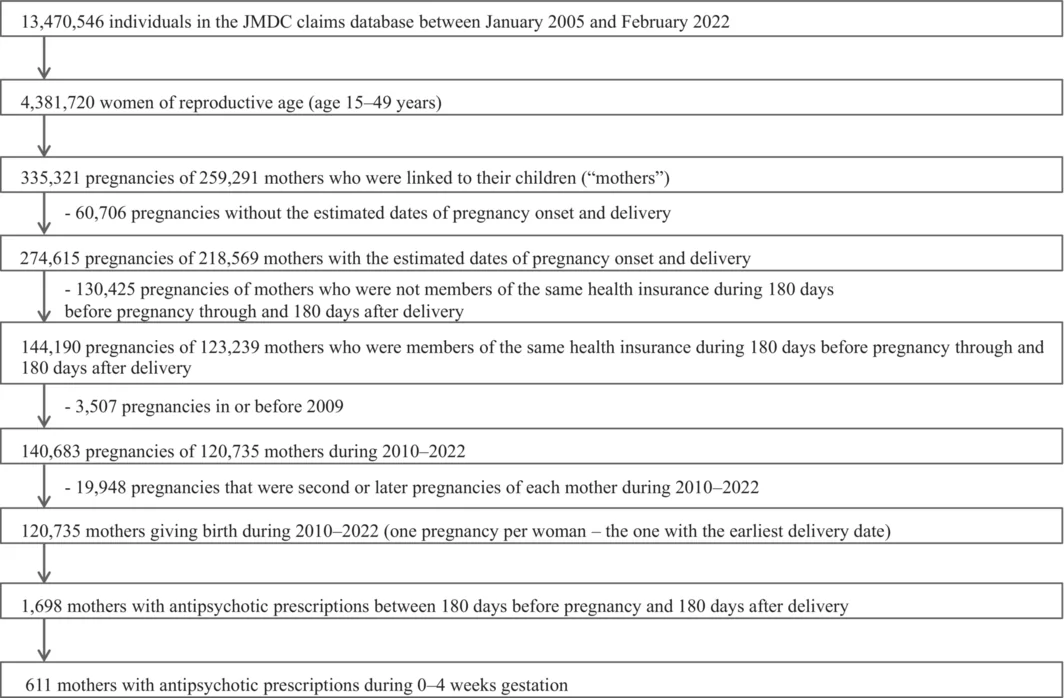
Flow chart of the study population. Selection of women from the JMDC claims database, including the identification of mothers and the application of the eligibility criteria for perinatal analysis.

Antipsychotics were identified by their non‐proprietary names; those evaluated are listed in Table S1, including all antipsychotics approved in Japan at the time of prescription. The dispensing date was preferentially used to estimate the timing of exposure; if unavailable, the hospitalization date was used because it was the only specific date available for drug data. With the month and year available for each claim, drugs were assigned to the 15th of the month if the dispensing or hospitalization date was unavailable. The days of supply for each prescription were considered to determine the exposure timing. Antipsychotics were classified according to their route of administration and generic names. As the only indications for injectable prochlorperazine in Japan are “preoperative and postoperative nausea and vomiting”, it was excluded from the analysis [23].

Antipsychotic prescription prevalence was determined 180 days before pregnancy onset, during pregnancy, and 180 days postpartum. Prescription prevalence was also calculated for 180–91 days and 90–1 days before pregnancy, for each trimester of pregnancy, and for 1–90 days and 91–180 days postpartum. Overall prevalence was calculated, as well as for each class and individual molecule.

New antipsychotic prescriptions occurring during the pre‐pregnancy period through the postpartum period were also evaluated, defined as the first antipsychotic prescription identified after confirming that no antipsychotics were prescribed during the baseline period. Prior use was assessed during a baseline window of 180–91 days before pregnancy. Women who received antipsychotics during this baseline period were considered to have a history of antipsychotic use. New antipsychotic prescriptions were investigated between 91 and 1 days before pregnancy, in each trimester of pregnancy, 1–90 days postpartum, and 91–180 days postpartum.

To evaluate antipsychotic prescription continuation, discontinuation, and resumption after pregnancy confirmation, we defined 5–13 weeks of gestation as the period when pregnancy is typically recognized in clinical practice. Prescription status was evaluated from 0 to 4, 5 to 13, and 14 weeks of gestation to delivery. Antipsychotic prescription was considered “continued” if there was at least one prescription in each consecutive period, and “discontinued” if there were no prescriptions in the period following the period of antipsychotic prescriptions; prescriptions were considered “resumed” if there was a prescription after antipsychotic prescriptions were discontinued. Among those prescribed antipsychotics during 0–4 weeks of gestation, prescription status and baseline characteristics were categorized as continued, discontinued, or resumed from 5 to 13 weeks of gestation and from 14 weeks of gestation to delivery, respectively. Stratified analyses by major indications for antipsychotic treatment were based on psychiatric diagnoses recorded before 4 weeks of gestation using ICD‐10 codes. All data were analyzed using SAS version 9.4 (SAS Institute Inc., Cary, NC, USA).

## 3 Results

Among 13 470 546 individuals, 4 381 720 were of reproductive age (15–49 years); of these, 335 321 were linked to their children, and 120 735 mothers met the eligibility criteria (Figure 1). Delivery dates were estimated based on the earliest dates of selected diagnoses and surgical procedures for 63 331 mothers (52.5%), the earliest dates of any other delivery‐related entry for 9417 (7.8%), and the 15th day of the neonatal birth month for 47 987 (39.7%). A term of 294 days was uniformly assigned as the gestational period for 1822 mothers (1.5%) whose gestational periods exceeded this value.

For the 120 735 mothers, the mean age at delivery and the length of gestation were 32.5 (standard deviation [SD]: 4.6) years and 270.2 (SD: 13.3) days, respectively (Table 1). Among included mothers, 120 394 (99.7%) reached the third trimester (Table 2). Before the delivery date, 1033 (0.9%), 756 (0.6%), 3817 (3.2%), 4576 (3.8%), and 680 (0.6%) of mothers were diagnosed with schizophrenia (ICD‐10: F20), bipolar disorder (F31), depressive episode (F32), other anxiety disorders (F41), and reaction to severe stress and adjustment disorders (F43), respectively, before delivery (Table 1).

**TABLE 1:** Characteristics of 120,735 mothers with or without antipsychotic prescriptions during pregnancy.

	Mothers meeting eligibility criteria	Antipsychotic prescriptions during pregnancy	
		Without	With
	*N* = 120,735	*N* = 119,926	*N* = 809
	*n* (%)	*n* (%)	*n* (%)
Maternal age at delivery (years), mean (SD)	32.5 (4.6)	32.5 (4.6)	33.3 (4.6)
≦ 24 years	4980 (4.1)	4960 (4.1)	20 (2.5)
25–29 years	27,538 (22.8)	27,387 (22.8)	151 (18.7)
30–34 years	47,125 (39.0)	46,807 (39.0)	318 (39.3)
≧ 35 years	41,092 (34.0)	40,772 (34.0)	320 (39.6)
Delivery year			
2010	1787 (1.5)	1773 (1.5)	14 (1.7)
2011	3539 (2.9)	3521 (2.9)	18 (2.2)
2012	5061 (4.2)	5035 (4.2)	26 (3.2)
2013	6420 (5.3)	6387 (5.3)	33 (4.1)
2014	8737 (7.2)	8685 (7.2)	52 (6.4)
2015	10,101 (8.4)	10,048 (8.4)	53 (6.6)
2016	11,837 (9.8)	11,765 (9.8)	72 (8.9)
2017	14 090 (11.7)	13 981 (11.7)	109 (13.5)
2018	16,395 (13.6)	16,307 (13.6)	88 (10.9)
2019	16,742 (13.9)	16,633 (13.9)	109 (13.5)
2020	16 150 (13.4)	16 014 (13.4)	136 (16.8)
2021	9876 (8.2)	9777 (8.2)	99 (12.2)
Length of gestation at delivery (days), mean (SD)	270.2 (13.3)	270.2 (13.3)	268.7 (13.7)
Preterm birth	14 939 (12.4)	14,822 (12.4)	117 (14.5)
Caesarean section	28,458 (23.6)	28,191 (23.5)	267 (33.0)
Newborn sex			
Male	61,520 (51.0)	61,071 (50.9)	449 (55.5)
Female	59,215 (49.0)	58,855 (49.1)	360 (44.5)
ICD‐10 code: Diagnosis names (major indications for antipsychotic)			
F20: Schizophrenia	1033 (0.9)	526 (0.4)	507 (62.7)
F30: Manic episode	84 (0.1)	50 (0.0)	34 (4.2)
F31: Bipolar disorder	756 (0.6)	514 (0.4)	242 (29.9)
F32: Depressive episode	3817 (3.2)	3357 (2.8)	460 (56.9)
F33: Recurrent depressive disorder	28 (0.0)	21 (0.0)	7 (0.9)
F34: Persistent mood (affective) disorders	468 (0.4)	418 (0.3)	50 (6.2)
F38: Other mood (affective) disorders	6 (0.0)	5 (0.0)	1 (0.1)
F39: Unspecified mood (affective) disorder	26 (0.0)	20 (0.0)	6 (0.7)
F40: Phobic anxiety disorders	244 (0.2)	220 (0.2)	24 (3.0)
F41: Other anxiety disorders	4576 (3.8)	4324 (3.6)	252 (31.1)
F42: Obsessive‐compulsive disorder	222 (0.2)	180 (0.2)	42 (5.2)
F43: Reaction to severe stress, and adjustment disorders	680 (0.6)	630 (0.5)	50 (6.2)
F84: Pervasive developmental disorders	74 (0.1)	56 (0.0)	18 (2.2)
K25: Gastric ulcer	9520 (7.9)	9357 (7.8)	163 (20.1)
K26: Duodenal ulcer	334 (0.3)	331 (0.3)	3 (0.4)
K910: Postoperative nausea	74 (0.1)	74 (0.1)	0 (0.0)
O21: Excessive vomiting in pregnancy	19,071 (15.8)	18,831 (15.7)	240 (29.7)
R11: Nausea and vomiting	19,762 (16.4)	19,517 (16.3)	245 (30.3)
R066: Hiccup	3 (0.0)	3 (0.0)	0 (0.0)

Abbreviations: ICD‐10, International Classification of Diseases 10 Revision; SD, standard deviation.

**TABLE 2:** Oral antipsychotics prescribed 180 days before and during pregnancy and 180 days after delivery (*N* = 120 735).

	Before pregnancy			During pregnancy				Postpartum		
	Within −180 days	−180 to −91 days	−90 to −1 days	Any trimester	First trimester	Second trimester	Third trimester^a^	Within 180 days	1 to 90 days	91 to 180 days
	*n* (Per 10 000)	*n* (Per 10 000)	*n* (Per 10 000)	*n* (Per 10 000)	*n* (Per 10 000)	*n* (Per 10 000)	*n* (Per 10 000)	*n* (Per 10 000)	*n* (Per 10 000)	*n* (Per 10 000)
Any oral antipsychotics	962 (79.7)	815 (67.5)	791 (65.5)	764 (63.3)	652 (54.0)	411 (34.0)	422 (35.1)	1003 (83.1)	802 (66.4)	710 (58.8)
Any oral typical antipsychotics	354 (29.3)	269 (22.3)	244 (20.2)	193 (16.0)	141 (11.7)	68 (5.6)	72 (6.0)	421 (34.9)	324 (26.8)	218 (18.1)
Any oral atypical antipsychotics	695 (57.6)	613 (50.8)	605 (50.1)	631 (52.3)	549 (45.5)	373 (30.9)	372 (30.9)	677 (56.1)	537 (44.5)	557 (46.1)
Benzamides	208 (17.2)	147 (12.2)	131 (10.9)	89 (7.4)	54 (4.5)	19 (1.6)	31 (2.6)	301 (24.9)	239 (19.8)	129 (10.7)
Nemonapride	0 (0.0)	0 (0.0)	0 (0.0)	0 (0.0)	0 (0.0)	0 (0.0)	0 (0.0)	0 (0.0)	0 (0.0)	0 (0.0)
Sultopride	0 (0.0)	0 (0.0)	0 (0.0)	0 (0.0)	0 (0.0)	0 (0.0)	0 (0.0)	0 (0.0)	0 (0.0)	0 (0.0)
Sulpiride	206 (17.1)	145 (12.0)	130 (10.8)	87 (7.2)	53 (4.4)	17 (1.4)	30 (2.5)	300 (24.8)	238 (19.7)	128 (10.6)
Tiapride	2 (0.2)	2 (0.2)	1 (0.1)	2 (0.2)	1 (0.1)	2 (0.2)	1 (0.1)	1 (0.1)	1 (0.1)	1 (0.1)
Butyrophenones	25 (2.1)	24 (2.0)	18 (1.5)	23 (1.9)	18 (1.5)	11 (0.9)	9 (0.7)	19 (1.6)	15 (1.2)	14 (1.2)
Bromperidol	5 (0.4)	5 (0.4)	5 (0.4)	5 (0.4)	5 (0.4)	1 (0.1)	0 (0.0)	3 (0.2)	2 (0.2)	2 (0.2)
Haloperidol	20 (1.7)	19 (1.6)	13 (1.1)	18 (1.5)	13 (1.1)	10 (0.8)	9 (0.7)	16 (1.3)	13 (1.1)	12 (1.0)
Pimozide	0 (0.0)	0 (0.0)	0 (0.0)	0 (0.0)	0 (0.0)	0 (0.0)	0 (0.0)	0 (0.0)	0 (0.0)	0 (0.0)
Pipamperone	0 (0.0)	0 (0.0)	0 (0.0)	0 (0.0)	0 (0.0)	0 (0.0)	0 (0.0)	0 (0.0)	0 (0.0)	0 (0.0)
Spiperone	0 (0.0)	0 (0.0)	0 (0.0)	0 (0.0)	0 (0.0)	0 (0.0)	0 (0.0)	0 (0.0)	0 (0.0)	0 (0.0)
Timiperone	0 (0.0)	0 (0.0)	0 (0.0)	0 (0.0)	0 (0.0)	0 (0.0)	0 (0.0)	0 (0.0)	0 (0.0)	0 (0.0)
Iminodibenzyl	0 (0.0)	0 (0.0)	0 (0.0)	0 (0.0)	0 (0.0)	0 (0.0)	0 (0.0)	0 (0.0)	0 (0.0)	0 (0.0)
Clocapramine	0 (0.0)	0 (0.0)	0 (0.0)	0 (0.0)	0 (0.0)	0 (0.0)	0 (0.0)	0 (0.0)	0 (0.0)	0 (0.0)
Mosapramine	0 (0.0)	0 (0.0)	0 (0.0)	0 (0.0)	0 (0.0)	0 (0.0)	0 (0.0)	0 (0.0)	0 (0.0)	0 (0.0)
Indoles	0 (0.0)	0 (0.0)	0 (0.0)	0 (0.0)	0 (0.0)	0 (0.0)	0 (0.0)	0 (0.0)	0 (0.0)	0 (0.0)
Oxypertin	0 (0.0)	0 (0.0)	0 (0.0)	0 (0.0)	0 (0.0)	0 (0.0)	0 (0.0)	0 (0.0)	0 (0.0)	0 (0.0)
Phenothiazines	130 (10.8)	103 (8.5)	98 (8.1)	86 (7.1)	72 (6.0)	38 (3.1)	31 (2.6)	104 (8.6)	71 (5.9)	74 (6.1)
Carpipramine	0 (0.0)	0 (0.0)	0 (0.0)	0 (0.0)	0 (0.0)	0 (0.0)	0 (0.0)	0 (0.0)	0 (0.0)	0 (0.0)
Chlorpromazine	53 (4.4)	43 (3.6)	35 (2.9)	27 (2.2)	24 (2.0)	12 (1.0)	9 (0.7)	35 (2.9)	26 (2.2)	24 (2.0)
Fluphenazine	3 (0.2)	3 (0.2)	2 (0.2)	2 (0.2)	2 (0.2)	2 (0.2)	1 (0.1)	2 (0.2)	2 (0.2)	2 (0.2)
Levomepromazine	51 (4.2)	44 (3.6)	44 (3.6)	34 (2.8)	31 (2.6)	17 (1.4)	13 (1.1)	39 (3.2)	28 (2.3)	29 (2.4)
Perphenazine	14 (1.2)	12 (1.0)	11 (0.9)	5 (0.4)	4 (0.3)	3 (0.2)	3 (0.2)	9 (0.7)	6 (0.5)	9 (0.7)
Prochlorperazine	18 (1.5)	7 (0.6)	12 (1.0)	19 (1.6)	12 (1.0)	4 (0.3)	5 (0.4)	23 (1.9)	14 (1.2)	12 (1.0)
Propericiazine	1 (0.1)	0 (0.0)	1 (0.1)	1 (0.1)	1 (0.1)	1 (0.1)	1 (0.1)	3 (0.2)	1 (0.1)	3 (0.2)
Thioridazine	0 (0.0)	0 (0.0)	0 (0.0)	0 (0.0)	0 (0.0)	0 (0.0)	0 (0.0)	0 (0.0)	0 (0.0)	0 (0.0)
DPA	356 (29.5)	305 (25.3)	310 (25.7)	298 (24.7)	276 (22.9)	161 (13.3)	159 (13.2)	281 (23.3)	216 (17.9)	239 (19.8)
Aripiprazole	342 (28.3)	295 (24.4)	299 (24.8)	284 (23.5)	264 (21.9)	155 (12.8)	153 (12.7)	260 (21.5)	204 (16.9)	221 (18.3)
Brexpiprazole	20 (1.7)	15 (1.2)	16 (1.3)	17 (1.4)	15 (1.2)	8 (0.7)	7 (0.6)	28 (2.3)	16 (1.3)	24 (2.0)
MARTA	304 (25.2)	262 (21.7)	254 (21.0)	300 (24.8)	232 (19.2)	175 (14.5)	174 (14.5)	352 (29.2)	271 (22.4)	270 (22.4)
Asenapine	8 (0.7)	7 (0.6)	4 (0.3)	6 (0.5)	4 (0.3)	1 (0.1)	3 (0.2)	6 (0.5)	5 (0.4)	4 (0.3)
Clozapine	0 (0.0)	0 (0.0)	0 (0.0)	0 (0.0)	0 (0.0)	0 (0.0)	0 (0.0)	0 (0.0)	0 (0.0)	0 (0.0)
Olanzapine	146 (12.1)	121 (10.0)	118 (9.8)	140 (11.6)	105 (8.7)	87 (7.2)	78 (6.5)	189 (15.7)	140 (11.6)	147 (12.2)
Quetiapine	172 (14.2)	147 (12.2)	148 (12.3)	175 (14.5)	135 (11.2)	99 (8.2)	100 (8.3)	184 (15.2)	143 (11.8)	135 (11.2)
SDA	201 (16.6)	171 (14.2)	157 (13.0)	174 (14.4)	138 (11.4)	98 (8.1)	92 (7.6)	214 (17.7)	152 (12.6)	172 (14.2)
Blonanserin	33 (2.7)	28 (2.3)	26 (2.2)	29 (2.4)	26 (2.2)	18 (1.5)	12 (1.0)	34 (2.8)	23 (1.9)	32 (2.7)
Lurasidone	2 (0.2)	0 (0.0)	2 (0.2)	2 (0.2)	2 (0.2)	1 (0.1)	1 (0.1)	6 (0.5)	2 (0.2)	6 (0.5)
Paliperidone	13 (1.1)	12 (1.0)	7 (0.6)	7 (0.6)	6 (0.5)	7 (0.6)	6 (0.5)	11 (0.9)	9 (0.7)	9 (0.7)
Perospirone	30 (2.5)	22 (1.8)	27 (2.2)	24 (2.0)	22 (1.8)	13 (1.1)	12 (1.0)	26 (2.2)	19 (1.6)	19 (1.6)
Risperidone	132 (10.9)	112 (9.3)	97 (8.0)	122 (10.1)	84 (7.0)	65 (5.4)	62 (5.1)	153 (12.7)	107 (8.9)	115 (9.5)
Zotepine^b^	7 (0.6)	7 (0.6)	5 (0.4)	4 (0.3)	4 (0.3)	1 (0.1)	1 (0.1)	8 (0.7)	5 (0.4)	6 (0.5)

Abbreviations: DPA, Dopamine partial agonist; MARTA, Multi‐acting receptor targeted antipsychotic; SDA, Serotonin‐dopamine antagonist.

^a^ Denominator of percentages is the number of pregnant women who reached third trimester (*n* = 120 394).

^b^ Zotepine is counted as a typical antipsychotic drug in the Drug class and as an SDA in the Drug subclass.

There were 119 451 records of antipsychotic prescriptions among the 120 735 mothers. Exposure timing was evaluated from 117 705 (98.5%) and 447 (0.4%) records based on dispensing and hospitalization dates, respectively. For the remaining 1299 records (1.1%), the 15th day of the month and year recorded for each claim were used. A total of 1698 mothers (140.6/10 000 deliveries) were prescribed antipsychotics at least once between 180 days before pregnancy onset and 180 days postpartum and were included in the analyses (Figure 1 and Table S2).

Compared with the pre‐pregnancy period—during which antipsychotics were prescribed to 818 mothers (67.8/10 000 deliveries) between 180 and 91 days before pregnancy and to 795 mothers (65.8/10 000 deliveries) between 90 and 1 days before pregnancy—prescription prevalence decreased during pregnancy (Table S3 and Figure S2). Antipsychotic prescriptions decreased in 656 mothers (54.3/10 000 deliveries) in the first trimester and 415 mothers (34.4/10 000 deliveries) in the second trimester. In contrast, prevalence increased postpartum, with antipsychotics prescribed to 820 mothers (67.9/10 000 deliveries) at 1–90 days postpartum and 714 (59.1/10 000 deliveries) at 91–180 days postpartum.

The most frequently prescribed oral antipsychotics before pregnancy were aripiprazole (28.3/10 000 deliveries), sulpiride (17.1/10 000 deliveries), and quetiapine (14.2/10 000 deliveries) (Table 2). During pregnancy, aripiprazole (23.5/10 000 deliveries), quetiapine (14.5/10 000 deliveries), and olanzapine (11.6/10 000 deliveries) were most common, while sulpiride (24.8/10 000 deliveries), aripiprazole (21.5/10 000 deliveries), and olanzapine (15.7/10 000 deliveries) were most common postpartum.

Haloperidol was the most frequently prescribed injectable antipsychotic before pregnancy (0.9/10 000 deliveries), followed by aripiprazole long‐acting injectable (LAI; 0.3/10 000 deliveries) and perphenazine (0.2/10 000 deliveries) (Table 3). During pregnancy, chlorpromazine (3.1/10 000 deliveries), haloperidol (1.2/10 000 deliveries), and aripiprazole LAI (0.3/10 000 deliveries) were the three most common injectable formulations, whereas haloperidol (2.8/10 000 deliveries), levomepromazine (0.7/10 000 deliveries), and aripiprazole LAI (0.4/10 000 deliveries) were the most frequently used postpartum. LAI antipsychotics were prescribed much less frequently than short‐acting injectable formulations throughout the study period (Table 3).

**TABLE 3:** Injectable antipsychotics prescribed 180 days before and during pregnancy and 180 days after delivery, excluding injectable prochlorperazine (*N* = 120 735).

	Before pregnancy			During pregnancy				Postpartum		
	Within −180 days	−180 to −91 days	−90 to −1 days	Any trimester	First trimester	Second trimester	Third trimester^a^	Within 180 days	1 to 90 days	91 to 180 days
	*n* (Per 10 000)	*n* (Per 10 000)	*n* (Per 10 000)	*n* (Per 10 000)	*n* (Per 10 000)	*n* (Per 10 000)	*n* (Per 10 000)	*n* (Per 10 000)	*n* (Per 10 000)	*n* (Per 10 000)
Any injectable antipsychotics^b^	20 (1.7)	11 (0.9)	14 (1.2)	60 (5.0)	8 (0.7)	11 (0.9)	51 (4.2)	52 (4.3)	40 (3.3)	21 (1.7)
Any injectable typical antipsychotics	14 (1.2)	6 (0.5)	9 (0.7)	54 (4.5)	3 (0.2)	6 (0.5)	47 (3.9)	47 (3.9)	36 (3.0)	14 (1.2)
Any injectable atypical antipsychotics	7 (0.6)	6 (0.5)	5 (0.4)	6 (0.5)	5 (0.4)	5 (0.4)	4 (0.3)	9 (0.7)	7 (0.6)	8 (0.7)
Benzamides^c^	1 (0.1)	1 (0.1)	1 (0.1)	0 (0.0)	0 (0.0)	0 (0.0)	0 (0.0)	1 (0.1)	0 (0.0)	1 (0.1)
Sulpiridel^c^	1 (0.1)	1 (0.1)	1 (0.1)	0 (0.0)	0 (0.0)	0 (0.0)	0 (0.0)	1 (0.1)	0 (0.0)	1 (0.1)
Butyrophenones^b^	11 (0.9)	5 (0.4)	6 (0.5)	15 (1.2)	3 (0.2)	4 (0.3)	10 (0.8)	35 (2.9)	27 (2.2)	10 (0.8)
Haloperidol^c^	11 (0.9)	5 (0.4)	6 (0.5)	14 (1.2)	3 (0.2)	3 (0.2)	9 (0.7)	34 (2.8)	26 (2.2)	9 (0.7)
Haloperidol^d^	0 (0.0)	0 (0.0)	0 (0.0)	1 (0.1)	0 (0.0)	1 (0.1)	1 (0.1)	2 (0.2)	1 (0.1)	2 (0.2)
Timiperone^c^	0 (0.0)	0 (0.0)	0 (0.0)	0 (0.0)	0 (0.0)	0 (0.0)	0 (0.0)	0 (0.0)	0 (0.0)	0 (0.0)
Phenothiazines^b^	2 (0.2)	0 (0.0)	2 (0.2)	40 (3.3)	0 (0.0)	2 (0.2)	38 (3.2)	14 (1.2)	11 (0.9)	4 (0.3)
Chlorpromazine^c^	0 (0.0)	0 (0.0)	0 (0.0)	38 (3.1)	0 (0.0)	2 (0.2)	36 (3.0)	4 (0.3)	4 (0.3)	0 (0.0)
Fluphenazine^d^	0 (0.0)	0 (0.0)	0 (0.0)	0 (0.0)	0 (0.0)	0 (0.0)	0 (0.0)	1 (0.1)	0 (0.0)	1 (0.1)
Levomepromazine^c^	0 (0.0)	0 (0.0)	0 (0.0)	2 (0.2)	0 (0.0)	1 (0.1)	1 (0.1)	8 (0.7)	6 (0.5)	3 (0.2)
Perphenazine^c^	2 (0.2)	0 (0.0)	2 (0.2)	1 (0.1)	0 (0.0)	0 (0.0)	1 (0.1)	1 (0.1)	1 (0.1)	0 (0.0)
DPA^d^	4 (0.3)	3 (0.2)	4 (0.3)	4 (0.3)	4 (0.3)	3 (0.2)	3 (0.2)	5 (0.4)	5 (0.4)	5 (0.4)
Aripiprazole^d^	4 (0.3)	3 (0.2)	4 (0.3)	4 (0.3)	4 (0.3)	3 (0.2)	3 (0.2)	5 (0.4)	5 (0.4)	5 (0.4)
MARTA^c^	1 (0.1)	1 (0.1)	0 (0.0)	1 (0.1)	0 (0.0)	1 (0.1)	0 (0.0)	4 (0.3)	2 (0.2)	3 (0.2)
Olanzapine^c^	1 (0.1)	1 (0.1)	0 (0.0)	1 (0.1)	0 (0.0)	1 (0.1)	0 (0.0)	4 (0.3)	2 (0.2)	3 (0.2)
SDA^d^	2 (0.2)	2 (0.2)	1 (0.1)	1 (0.1)	1 (0.1)	1 (0.1)	1 (0.1)	2 (0.2)	1 (0.1)	2 (0.2)
Paliperidone^d^	1 (0.1)	1 (0.1)	1 (0.1)	1 (0.1)	1 (0.1)	1 (0.1)	1 (0.1)	2 (0.2)	1 (0.1)	2 (0.2)
Risperidone^d^	1 (0.1)	1 (0.1)	0 (0.0)	0 (0.0)	0 (0.0)	0 (0.0)	0 (0.0)	0 (0.0)	0 (0.0)	0 (0.0)
Classification by injectable formulation types^e^										
Short‐acting injectable formulations										
Chlorpromazine	0 (0.0)	0 (0.0)	0 (0.0)	38 (3.1)	0 (0.0)	2 (0.2)	36 (3.0)	4 (0.3)	4 (0.3)	0 (0.0)
Haloperidol	11 (0.9)	5 (0.4)	6 (0.5)	14 (1.2)	3 (0.2)	3 (0.2)	9 (0.7)	34 (2.8)	26 (2.2)	9 (0.7)
Levomepromazine	0 (0.0)	0 (0.0)	0 (0.0)	2 (0.2)	0 (0.0)	1 (0.1)	1 (0.1)	8 (0.7)	6 (0.5)	3 (0.2)
Olanzapine	1 (0.1)	1 (0.1)	0 (0.0)	1 (0.1)	0 (0.0)	1 (0.1)	0 (0.0)	4 (0.3)	2 (0.2)	3 (0.2)
Perphenazine	2 (0.2)	0 (0.0)	2 (0.2)	1 (0.1)	0 (0.0)	0 (0.0)	1 (0.1)	1 (0.1)	1 (0.1)	0 (0.0)
Sulpiride	1 (0.1)	1 (0.1)	1 (0.1)	0 (0.0)	0 (0.0)	0 (0.0)	0 (0.0)	1 (0.1)	0 (0.0)	1 (0.1)
Long‐acting injectable formulations										
Aripiprazole	4 (0.3)	3 (0.2)	4 (0.3)	4 (0.3)	4 (0.3)	3 (0.2)	3 (0.2)	5 (0.4)	5 (0.4)	5 (0.4)
Fluphenazine	0 (0.0)	0 (0.0)	0 (0.0)	0 (0.0)	0 (0.0)	0 (0.0)	0 (0.0)	1 (0.1)	0 (0.0)	1 (0.1)
Haloperidol	0 (0.0)	0 (0.0)	0 (0.0)	1 (0.1)	0 (0.0)	1 (0.1)	1 (0.1)	2 (0.2)	1 (0.1)	2 (0.2)

Abbreviations: DPA, dopamine partial agonist; MARTA, multi‐acting receptor‐targeted antipsychotic; SDA, serotonin–dopamine antagonist.

^a^ Denominator of percentages is the number of pregnant women who reached third trimester (*n* = 120 394).

^b^ Any injectable antipsychotics include both short‐acting injectable and long‐acting injectable formulations.

^c^ Short‐acting injectable formulations.

^d^ Long‐acting injectable formulations.

^e^ The sub‐section “Classification by injectable formulation type” lists only those antipsychotic drugs for which at least one prescription was identified during any of the analyzed periods.

Transdermal antipsychotic use was rare; only blonanserin patches were prescribed, with no use before pregnancy, 0.1/10 000 deliveries during pregnancy, and 0.2/10 000 deliveries postpartum (Table S4). Oral and injectable haloperidol, which is contraindicated during pregnancy in Japan, was prescribed to some women. Any oral antipsychotic prescription during pregnancy decreased from the first to the second trimester, leveled off during the second to the third trimester, and returned to near pre‐pregnancy levels postpartum (Figure S2 and Table 2). In contrast, the number of injectable antipsychotic prescriptions was higher in the third trimester.

Trends in new antipsychotic prescriptions from 180 days of pregnancy to 180 days postpartum are shown in Figure 2. New antipsychotic prescriptions were low during pregnancy but high postpartum; a high number of new antipsychotics were prescribed 1–90 days postpartum. Although the number of antipsychotic prescriptions in the pre‐pregnancy and postpartum periods appeared to be equal, the number of antipsychotic prescriptions postpartum included many new prescriptions. Most new antipsychotic prescriptions in the postpartum period were oral sulpiride (Table S5).

**FIGURE 2:**
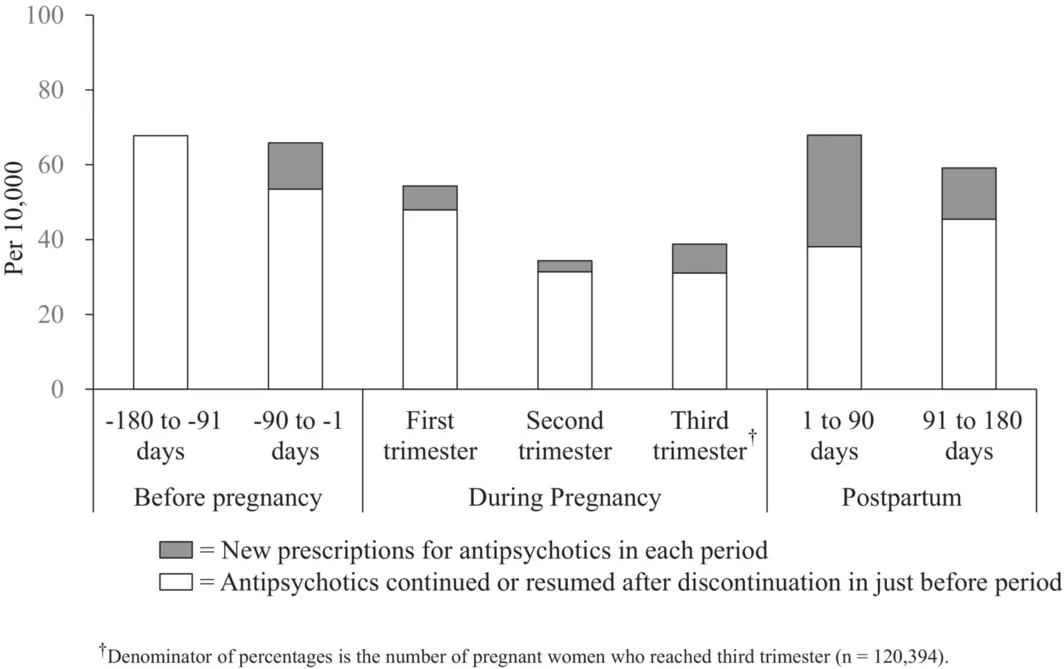
New prescriptions for antipsychotics from 90 days before pregnancy to 180 days postpartum. Antipsychotic prescription patterns before pregnancy, during pregnancy, and in the postpartum period, including new prescriptions. New prescriptions were defined as the first antipsychotic prescription after the baseline period without antipsychotic use (180–91 days before pregnancy).

Figure 3 shows the trend in antipsychotic prescriptions at 5 weeks of gestation after pregnancy confirmation. A total of 611 pregnant women received antipsychotic prescriptions at 0–4 weeks of gestation. In total, 482 women (78.9%) continued antipsychotic prescriptions during 5–13 weeks of gestation, while 129 (21.1%) discontinued; 334 (54.7%) continued until delivery, while 110 (18.0%) discontinued before delivery. Among 334 women whose prescriptions were continued until delivery, schizophrenia was the most common diagnosis before 4 weeks of gestation (267, 79.9%) (Table 4). Among 110 women who discontinued, depressive episodes were the most frequent diagnosis (82, 74.5%). Among those who received antipsychotic prescriptions at 0–4 weeks of gestation, evaluation of major indications for antipsychotic treatment showed that continuation rates until delivery were highest among those with schizophrenia (63.6%), followed by bipolar disorder (51.0%) (Figure 4).

**FIGURE 3:**
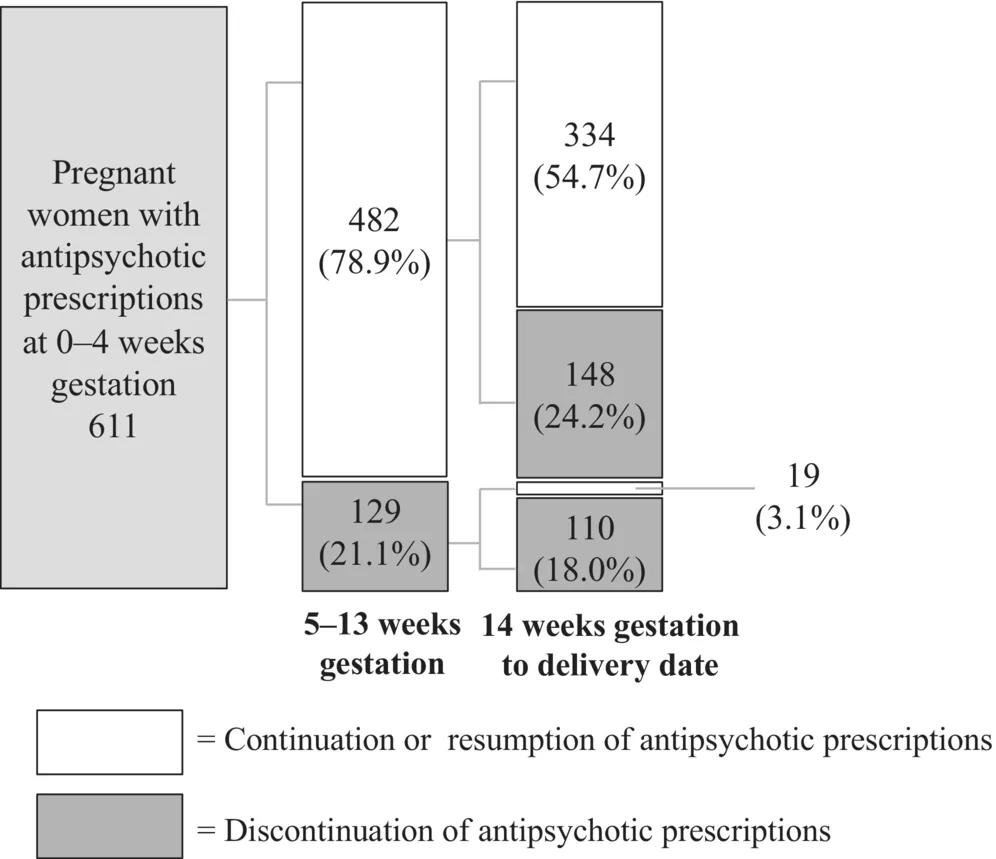
Antipsychotic prescriptions status after pregnancy confirmation. Patterns of continuation, discontinuation, and resumption of antipsychotic prescriptions after pregnancy confirmation, with the prescription status evaluated at 5 weeks of gestation.

**TABLE 4:** Characteristics of 611 women prescribed antipsychotics during 0–4 weeks gestation, excluding injectable prochlorperazine (*N* = 120 735).

	Women with antipsychotic prescriptions during 0–4 weeks gestation	Women prescribed antipsychotics during 0–4 weeks of gestation: prescription status from 5 weeks of gestation to delivery date			
		Continued antipsychotic prescriptions from 5 weeks gestation to delivery date	Continued antipsychotic prescriptions during 5–13 weeks gestation, then discontinued from 14 weeks gestation to delivery date	Discontinued antipsychotic prescriptions during 5–13 weeks gestation, then resumed from 14 weeks gestation to delivery date	Discontinued antipsychotic prescriptions from 5 weeks gestation to delivery date
	*N* = 611	*N* = 334	*N* = 148	*N* = 19	*N* = 110
	*n* (%)	*n* (%)	*n* (%)	*n* (%)	*n* (%)
Maternal age at delivery (years), mean (SD)	33.3 (4.6)	34.1 (4.6)	31.9 (4.5)	33.8 (4.5)	32.8 (4.5)
≦ 24 years	15 (2.5)	4 (1.2)	9 (6.1)	0 (0.0)	2 (1.8)
25–29 years	111 (18.2)	51 (15.3)	34 (23)	3 (15.8)	23 (20.9)
30–34 years	242 (39.6)	127 (38.0)	63 (42.6)	7 (36.8)	45 (40.9)
≧ 35 years	243 (39.8)	152 (45.5)	42 (28.4)	9 (47.4)	40 (36.4)
Delivery year					
2010	10 (1.6)	4 (1.2)	4 (2.7)	0 (0.0)	2 (1.8)
2011	10 (1.6)	4 (1.2)	2 (1.4)	1 (5.3)	3 (2.7)
2012	13 (2.1)	6 (1.8)	3 (2.0)	0 (0.0)	4 (3.6)
2013	24 (3.9)	12 (3.6)	7 (4.7)	0 (0.0)	5 (4.5)
2014	38 (6.2)	19 (5.7)	14 (9.5)	1 (5.3)	4 (3.6)
2015	44 (7.2)	25 (7.5)	11 (7.4)	1 (5.3)	7 (6.4)
2016	53 (8.7)	30 (9.0)	14 (9.5)	1 (5.3)	8 (7.3)
2017	82 (13.4)	39 (11.7)	24 (16.2)	3 (15.8)	16 (14.5)
2018	68 (11.1)	41 (12.3)	17 (11.5)	1 (5.3)	9 (8.2)
2019	86 (14.1)	51 (15.3)	16 (10.8)	3 (15.8)	16 (14.5)
2020	105 (17.2)	58 (17.4)	22 (14.9)	5 (26.3)	20 (18.2)
2021	78 (12.8)	45 (13.5)	14 (9.5)	3 (15.8)	16 (14.5)
Length of gestation at delivery (days), mean (SD)	268.3 (14.6)	267.6 (15.8)	269.5 (13.7)	268.3 (15.4)	269.1 (11.2)
Preterm birth	92 (15.1)	56 (16.8)	19 (12.8)	3 (15.8)	14 (12.7)
Caesarean section	183 (30.0)	117 (35.0)	40 (27.0)	5 (26.3)	21 (19.1)
Newborn sex					
Male	335 (54.8)	180 (53.9)	84 (56.8)	15 (78.9)	56 (50.9)
Female	276 (45.2)	154 (46.1)	64 (43.2)	4 (21.1)	54 (49.1)
ICD‐10 code: Diagnosis names					
F20: Schizophrenia	420 (68.7)	267 (79.9)	84 (56.8)	15 (78.9)	54 (49.1)
F31: Bipolar disorder	194 (31.8)	99 (29.6)	59 (39.9)	7 (36.8)	29 (26.4)
F30: Manic episode	30 (4.9)	21 (6.3)	4 (2.7)	1 (5.3)	4 (3.6)
F32: Depressive episode	355 (58.1)	169 (50.6)	91 (61.5)	13 (68.4)	82 (74.5)
F33: Recurrent depressive disorder	1 (0.2)	1 (0.3)	0 (0.0)	0 (0.0)	0 (0.0)
F34: Persistent mood (affective) disorders	36 (5.9)	18 (5.4)	11 (7.4)	1 (5.3)	6 (5.5)
F38: Other mood (affective) disorders	0 (0.0)	0 (0.0)	0 (0.0)	0 (0.0)	0 (0.0)
F39: Unspecified mood (affective) disorder	4 (0.7)	3 (0.9)	1 (0.7)	0 (0.0)	0 (0.0)
F40: Phobic anxiety disorders	17 (2.8)	5 (1.5)	3 (2.0)	0 (0.0)	0 (0.0)
F41: Other anxiety disorders	164 (26.8)	83 (24.9)	43 (29.1)	7 (36.8)	31 (28.2)
F42: Obsessive‐compulsive disorder	28 (4.6)	15 (4.5)	7 (4.7)	0 (0.0)	6 (5.5)
F43: Reaction to severe stress, and adjustment disorders	38 (6.2)	13 (3.9)	11 (7.4)	2 (10.5)	12 (10.9)
F84: Pervasive developmental disorders	14 (2.3)	6 (1.8)	6 (4.1)	0 (0.0)	2 (1.8)
R066: Hiccup	0 (0.0)	0 (0.0)	0 (0.0)	0 (0.0)	0 (0.0)
R11: Nausea and vomiting	140 (22.9)	68 (20.4)	46 (31.1)	4 (21.1)	22 (20.0)
K25: Gastric ulcer	103 (16.9)	55 (16.5)	28 (18.9)	1 (5.3)	19 (17.3)
K26: Duodenal ulcer	3 (0.5)	2 (0.6)	0 (0.0)	0 (0.0)	1 (0.9)
K910: Postoperative nausea	0 (0.0)	0 (0.0)	0 (0.0)	0 (0.0)	0 (0.0)
O21: Excessive vomiting in pregnancy	23 (3.8)	6 (1.8)	7 (4.7)	1 (5.3)	9 (8.2)

Abbreviation: ICD‐10, International Classification of Diseases 10 Revision.

**FIGURE 4:**
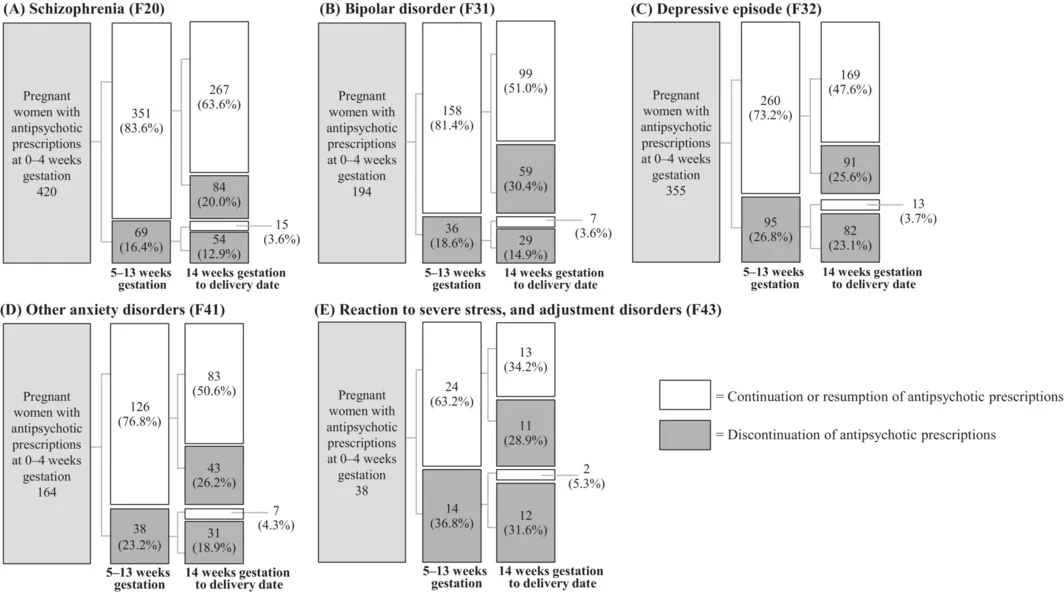
Antipsychotic prescription status after pregnancy confirmation by major indications for antipsychotic treatment. (A) Schizophrenia (F20); (B) Bipolar disorder (F31); (C) Depressive episode (F32); (D) Other anxiety disorders (F41); and (E) Reaction to severe stress, and adjustment disorders (F43).

## 4 Discussion

This is the first evaluation of antipsychotic prescriptions before, during, and after pregnancy in Japan using a large claims database. Among 120 735 mothers, 809 (67.0/10 000 deliveries) were prescribed antipsychotics at least once during pregnancy (Table S3). This prevalence was higher than that reported in European cohorts (0.1%–0.3%) but lower than that reported in the United States (0.7%–1.1%) [24, 25, 26, 27].

Schizophrenia, bipolar disorder, and depressive episodes were common diagnoses before delivery among pregnant women prescribed antipsychotics (Table 1). A survey of pregnant women prescribed antipsychotics in the United States also reported depression, bipolar disorder, and schizophrenia as the most common diagnoses [26]. Although the proportions differed between studies, these findings suggest that antipsychotics are prescribed perinatally across similar major psychiatric conditions [27]. The Guideline for Pharmacological Therapy of Schizophrenia 2022 in Japan suggests that antipsychotic treatment during pregnancy can reduce relapse and hospitalization and recommends continuation when clinically indicated, despite potential adverse events [10].

Oral antipsychotics were prescribed more frequently than injectable or transdermal formulations. The most frequently prescribed oral antipsychotics before pregnancy were aripiprazole, sulpiride, and quetiapine; aripiprazole, quetiapine, and olanzapine were most common during pregnancy, while sulpiride, aripiprazole, and olanzapine were most frequently prescribed postpartum. A multinational study reported that quetiapine, olanzapine, aripiprazole, and prochlorperazine are commonly prescribed [28], aligning with our findings. In Japan, a claims‐based study of patients with schizophrenia reported frequent use of aripiprazole, risperidone, olanzapine, and quetiapine [29].

Although our study was not limited to patients with schizophrenia, a similar preference for atypical antipsychotics has been observed in the perinatal population. Injectable or transdermal antipsychotics were rarely administered; LAI antipsychotics were prescribed in ≤ 0.4/10 000 deliveries across all periods, indicating that oral formulations remain the mainstay of perinatal antipsychotic treatment in Japan. LAI antipsychotics were prescribed much less frequently than short‐acting injectable formulations throughout the study period. Although the number of LAI antipsychotic prescriptions was limited, detailed discussion of their prescribing patterns was difficult. However, their prescribing frequency changed little from before pregnancy through the postpartum period, suggesting that they may have been used as maintenance treatment in a similar manner to oral formulations. In contrast, short‐acting injectable formulations were prescribed less frequently during the first and second trimesters but increased during the third trimester and the postpartum period, suggesting that they may have been used primarily for the management of acute symptoms. The limited use of LAI antipsychotics may be attributed to their higher cost and the relative difficulty of immediate dose adjustment compared with oral formulations, as well as to the limited safety data available during pregnancy [30, 31, 32]. These considerations may be particularly relevant during the perinatal period, when psychiatric symptoms and treatment needs can change over time, and may have contributed to the infrequent use of LAI antipsychotics in the present study. Although contraindicated during pregnancy in Japan based on case reports of limb malformations and teratogenic findings in animal studies, oral and injectable haloperidol are prescribed to some women [33]; bromperidol is similarly contraindicated [34]. However, haloperidol is not contraindicated during pregnancy in the United States [35, 36]. Our results indicate that despite domestic contraindications, haloperidol continues to be prescribed to some pregnant women in Japan, highlighting the need for further accumulation of safety data and careful risk–benefit evaluation in clinical practice.

New prescriptions of antipsychotics were uncommon during pregnancy, but increased markedly during the postpartum period, particularly within 90 days after delivery. Although the overall number of prescriptions was similar before pregnancy and after delivery, the postpartum period included a larger proportion of new users. Most new postpartum prescriptions involved oral sulpiride, possibly reflecting its off‐label use as a galactagogue rather than for primary psychiatric indications [37, 38, 39, 40].

To evaluate prescription behavior after pregnancy confirmation, continuation and discontinuation were assessed from early gestation through delivery. Among those prescribed antipsychotics during 0–4 weeks of gestation, 78.9% continued treatment during 5–13 weeks, whereas 21.1% discontinued; by delivery, 54.7% continued treatment and 18.0% had discontinued. Previous Japanese data on antipsychotic continuation during pregnancy are lacking, whereas U.S. studies report continuation rates of approximately 64.6% [41]. The lower continuation rates in Japan may reflect differences in prescription practices and concerns regarding fetal safety. In a previous report, 45.5% of women who reduced or discontinued antipsychotic medication after becoming pregnant had unstable psychiatric symptoms requiring treatment adjustments before delivery [42]. In our study, it was not possible to determine whether antipsychotic prescriptions were discontinued after pregnancy confirmation because treatment was judged unnecessary or despite the continuing need for treatment. Some women resumed antipsychotic treatment after discontinuation, suggesting that symptom relapse or worsening may have necessitated the resumption of treatment before delivery. These findings raise the possibility that some women who discontinue treatment require antipsychotic therapy during pregnancy.

Continuation patterns differed according to diagnosis. Among those who continued treatment until delivery, schizophrenia was the most common diagnosis, whereas depressive episodes predominated among those who discontinued. Continuation rates declined after the first trimester across diagnostic groups; however, those with schizophrenia consistently showed the highest continuation rates. These findings align with the Perinatal Mental Health Consensus Guide 2023, as well as its earlier edition published in 2017, both of which recommend antipsychotic continuation in those with schizophrenia because of the high relapse risk [8]. For depressive and anxiety disorders, clinicians are more likely to discontinue antipsychotics after pregnancy confirmation [12]. In such cases, symptoms may instead be managed with other psychotropic medications such as antidepressants, which have been continued during pregnancy in recent years [4]. Although continuation rates were relatively higher in patients with schizophrenia and bipolar disorder, nearly half of those with bipolar disorder and one‐third of those with schizophrenia discontinued treatment before delivery. Discontinuation among those with these conditions warrants particular attention because maintenance therapy interruption in these conditions is strongly associated with relapse and hospitalization, particularly prenatally, when the risk of illness exacerbation may be high [7].

This study has some limitations. First, because we used the JMDC claims database, it was not possible to evaluate disease severity, detailed clinical indications for each prescription, or medication adherence, or to account for important sociodemographic or clinical factors associated with antenatal mental health. In claims data, dispensed medications do not necessarily indicate actual medication use; continuation may have been overestimated if medication was discontinued despite the recorded dispensing. Second, the generalizability of our findings to the entire Japanese perinatal population may be limited in terms of both population coverage and representativeness. Regarding population coverage, the 140 683 pregnancies identified in this study account for approximately 1.1% of the total 12 328 734 live births in Japan during the study period (2010–2022), according to the Vital Statistics of the Ministry of Health, Labour and Welfare [43]. Although this coverage was limited, a previous assessment reported that mother–infant pairs identified in the JMDC database accounted for approximately 2% of live births in Japan, suggesting that similar population coverage was observed in that study [44]. Furthermore, regarding representativeness, the JMDC claims database primarily captures employees of relatively large companies and their dependents. In addition, because mothers and infants can only be linked if they are enrolled under the same health insurer, our study population was restricted to such cases. These characteristics may introduce some degree of selection bias. Nevertheless, the database includes a large population drawn from across Japan and has been widely used in pharmacoepidemiological studies in Japan [4, 12, 13, 15, 16]. A recent assessment also reported that mother–infant pairs identified in the JMDC database may be suitable for descriptive analyses [44]. Therefore, the present findings are likely to reflect prescribing patterns among a substantial proportion of pregnant women receiving routine clinical care. The absence of clozapine prescriptions in our study should be interpreted with caution. Although clozapine use during pregnancy has been reported in a few cases in Japan [45, 46], it appears to be extremely limited. Clozapine is approved only for treatment‐resistant schizophrenia in Japan. Previous studies have reported that clozapine use in Japan remains relatively limited compared with many other countries [47]. Moreover, clozapine is subject to strict prescribing and monitoring requirements [48]. These factors may contribute to the limited use of clozapine compared with other antipsychotics. Given the rarity of clozapine exposure during pregnancy, its absence in the present cohort may reflect the low frequency of exposure rather than incomplete capture within the database. Accordingly, caution is warranted when generalizing our findings to women with severe psychiatric disorders requiring highly specialized treatment. Third, drug exposure timing may have been misclassified because the dates of delivery and drug dispensing were sometimes estimated [13, 19, 21]; however, previous studies using the same pregnancy estimation algorithm reported consistent prescription patterns [12], suggesting that any misclassification was unlikely to have materially affected our results, particularly because dispensing dates were available for most prescriptions. Fourth, as our study design relied on the above‐mentioned linkage between mothers and liveborn children, mothers whose pregnancies ended in an abortion or stillbirth could not be evaluated. Further studies using other data sources and newly developed algorithms to identify miscarriages, stillbirths, and abortions are needed to address these limitations. Finally, prescribing prevalence was expressed as the number of women prescribed antipsychotics per 10 000 deliveries within each period rather than as person‐time–based rates. Although the lengths of the evaluated periods were broadly similar (approximately 90–100 days), minor differences in period duration may have influenced the absolute prevalence estimates; however, the overall temporal trends are unlikely to have been materially affected.

In conclusion, various antipsychotics are prescribed for pregnant and postpartum women in Japan. Approximately half of those prescribed antipsychotics around pregnancy confirmation discontinued treatment during pregnancy, while postpartum increases largely reflected new prescriptions. Schizophrenia was associated with the highest continuation rates, whereas depressive episodes were associated with lower continuation rates. This study provides the first comprehensive description of perinatal antipsychotic prescription patterns in Japan and offers insights that may inform perinatal mental health care beyond the Japanese setting, as well as epidemiological information to help characterize real‐world prescription trends. Additional evidence is needed to support risk–benefit assessments and shared decision‐making in perinatal mental healthcare.

## Author Contributions

**Ryo Obara:** writing – review and editing, conceptualization, formal analysis, funding acquisition, writing – original draft, methodology. **Takamasa Sakai:** writing – review and editing, methodology, conceptualization, formal analysis. **Tomofumi Ishikawa:** writing – review and editing, conceptualization, methodology. **Kei Morishita:** writing – review and editing. **Motohiko Adomi:** writing – review and editing. **Azusa Hara:** writing – review and editing. **Saya Kikuchi:** writing – review and editing. **Natsuko Kobayashi:** writing – review and editing. **Noriyuki Iwama:** writing – review and editing. **Genki Shinoda:** writing – review and editing. **Aoi Noda:** writing – review and editing. **Masatsugu Orui:** writing – review and editing. **Mami Ishikuro:** writing – review and editing. **Hiroaki Tomita:** writing – review and editing. **Nariyasu Mano:** writing – review and editing. **Shinichi Kuriyama:** writing – review and editing. **Taku Obara:** writing – review and editing, formal analysis, supervision, project administration, conceptualization, funding acquisition.

## Funding

This work was supported by grants from the Ministry of Health, Labour and Welfare of Japan (H23‐iyaku‐ippan‐006), the Japan Society for the Promotion of Science—Grant Number (JSPS KAKENHI; JP 25 K13625), and the Research Institute of Healthcare Data Science. The funders had no role in the study design, data analysis, or publication.

## Ethics Statement

The Institutional Review Board of Tohoku University School of Medicine approved this study on January 22, 2024 (registration number: 2023‐1‐808). The requirement for informed consent was waived.

## Conflicts of Interest

Tomofumi Ishikawa is an employee of Pfizer Research and Development in Japan. Tomofumi Ishikawa is also a research collaborator at Tohoku University and contributed to the present study independently of Pfizer R&D in Japan.

## Supporting information


**Figure S1:** Schematic illustration of the estimation of the dates of pregnancy onset and delivery based on the algorithm.
Pregnancy onset was estimated from gestational age specified in diagnosis records, whereas delivery was estimated from delivery‐related diagnosis, procedure, or drug records, or from the 15th day of the neonatal birth month when unavailable.
**Table S1:** Antipsychotics investigated in the current study.
**Table S2:** Antipsychotics prescribed between 180 days before pregnancy and 180 days after delivery (*N* = 120 735).
**Table S3:** Typical or atypical antipsychotics prescribed 180 days before and during pregnancy and 180 days after delivery, excluding injectable prochlorperazine (*N* = 120 735).
**Table S4:** The transdermal antipsychotics prescribed 180 days before and during pregnancy and 180 days after delivery (*N* = 120 735).
**Table S5:** Antipsychotics newly prescribed from 90 days before pregnancy to 180 days postpartum, excluding injectable prochlorperazine (*N* = 120 735).

**Figure S2:** Prescriptions for antipsychotics from 180 days before pregnancy to 180 days postpartum. Antipsychotic prescription patterns by formulation (oral, injectable, and transdermal) across the pre‐pregnancy, pregnancy, and postpartum periods.

## Data Availability

The data that supports the findings of this study are available in the Supporting Information of this article.
