# Supplementary material for: Antipsychotic Prescriptions for Prenatal and Postpartum Women in Japan: A Large Claims Database Study
Source: Pharmacol Res Perspect. 2026 Oct 28;14(6):e70336. doi: 10.1002/prp2.70336 (PMC13716938; doi:10.1002/prp2.70336)
Supplement: Supplementary file 1 [file PRP2-14-e70336-s002.docx]

**Supplementary Appendix**

[**Table S1.** Antipsychotics investigated in the current study	2](#_Toc226317177)

[**Table S2.** Antipsychotics prescribed between 180 days before pregnancy and 180 days after delivery (N=120,735).	3](#_Toc226317178)

[**Table S3.** Typical or atypical antipsychotics prescribed 180 days before and during pregnancy and 180 days after delivery, excluding injectable prochlorperazine (N=120,735).	4](#_Toc226317179)

[**Table S4.** The transdermal antipsychotics prescribed 180 days before and during pregnancy and 180 days after delivery (N=120,735).	4](#_Toc226317180)

[**Table S5.** Antipsychotics newly prescribed from 90 days before pregnancy to 180 days postpartum, excluding injectable prochlorperazine (N=120,735).	5](#_Toc226317181)

[**Estimation of pregnancy onset and delivery**	6](#_Toc226317182)

# **Table S1.** Antipsychotics investigated in the current study

| Drug class | Drug subclass | Nonproprietary name | WHO-ATC codes^a^ |
| --- | --- | --- | --- |
| Typical antipsychotic | Benzamide | Nemonapride | N05AX^b^ |
|  |  | Sulpiride | N05AL01 |
|  |  | Sultopride | N05AL02 |
|  |  | Tiapride | N05AL03 |
|  | Butyrophenone | Bromperidol | N05AD06 |
|  |  | Haloperidol | N05AD01 |
|  |  | Pimozide | N05AG02 |
|  |  | Pipamperone | N05AD05 |
|  |  | Spiperone | N05AX^b^ |
|  |  | Timiperone | N05AX^b^ |
|  | Iminodibenzyl | Clocapramine | N05AX^b^ |
|  |  | Mosapramine | N05AX10 |
|  | Indole | Oxypertin | N05AE01 |
|  | Phenothiazine | Carpipramine | N05AX^b^ |
|  |  | Chlorpromazine | N05AA01 |
|  |  | Fluphenazine | N05AB02 |
|  |  | Levomepromazine | N05AA02 |
|  |  | Perphenazine | N05AB03 |
|  |  | Prochlorperazine  (Excluding injectable drugs) | N05AB04 |
|  |  | Propericiazine | N05AC01 |
|  |  | Thioridazine | N05AC02 |
|  | Serotonin-dopamine antagonist | Zotepine | N05AX11 |
| Atypical antipsychotic | Dopamine partial agonist | Aripiprazole | N05AX12 |
|  |  | Brexpiprazole | N05AX16 |
|  | Multi-acting receptor-targeted antipsychotic | Asenapine | N05AH05 |
|  |  | Clozapine | N05AH02 |
|  |  | Olanzapine | N05AH03 |
|  |  | Quetiapine | N05AH04 |
|  | Serotonin-dopamine antagonist | Blonanserin | N05AX^b^ |
|  |  | Lurasidone | N05AE05 |
|  |  | Paliperidone | N05AX13 |
|  |  | Perospirone | N05AX^b^ |
|  |  | Risperidone | N05AX08 |

Abbreviations: ATC, Anatomical Therapeutic Chemical; WHO, World Health Organization.

^a^In this study, antipsychotics were identified by their nonproprietary names; however, the corresponding ATC codes are provided in this table.
^b^A specific code is unavailable for these drugs.

**Table S2.** Antipsychotics prescribed between 180 days before pregnancy and 180 days after delivery (N=120,735).

|  | n | (per10,000) |
| --- | --- | --- |
| Any antipsychotics | 1,698 | (140.6) |
| Any typical antipsychotics | 859 | (71.1) |
| Any atypical antipsychotics | 1,044 | (86.5) |
| Any oral antipsychotics | 1,633 | (135.3) |
| Oral typical antipsychotics | 775 | (64.2) |
| Oral atypical antipsychotics | 1,043 | (86.4) |
| Benzamides | 531 | (44.0) |
| Nemonapride | 0 | (0.0) |
| Sultopride | 0 | (0.0) |
| Sulpiride | 528 | (43.7) |
| Tiapride | 3 | (0.2) |
| Butyrophenones | 37 | (3.1) |
| Bromperidol | 6 | (0.5) |
| Haloperidol | 31 | (2.6) |
| Pimozide | 0 | (0.0) |
| Pipamperone | 0 | (0.0) |
| Spiperone | 0 | (0.0) |
| Timiperone | 0 | (0.0) |
| Iminodibenzyl | 0 | (0.0) |
| Clocapramine | 0 | (0.0) |
| Mosapramine | 0 | (0.0) |
| Indoles | 0 | (0.0) |
| Oxypertin | 0 | (0.0) |
| Phenothiazines | 228 | (18.9) |
| Carpipramine | 0 | (0.0) |
| Chlorpromazine | 83 | (6.9) |
| Fluphenazine | 4 | (0.3) |
| Levomepromazine | 77 | (6.4) |
| Perphenazine | 20 | (1.7) |
| Prochlorperazine | 56 | (4.6) |
| Propericiazine | 3 | (0.2) |
| Thioridazine | 0 | (0.0) |
| DPA | 482 | (39.9) |
| Aripiprazole | 457 | (37.9) |
| Brexpiprazole | 39 | (3.2) |
| MARTA | 563 | (46.6) |
| Asenapine | 16 | (1.3) |
| Clozapine | 0 | (0.0) |
| Olanzapine | 294 | (24.4) |
| Quetiapine | 316 | (26.2) |
| SDA | 361 | (29.9) |
| Blonanserin | 58 | (4.8) |
| Lurasidone | 8 | (0.7) |
| Paliperidone | 21 | (1.7) |
| Perospirone | 44 | (3.6) |
| Risperidone | 271 | (22.4) |
| Zotepine^†^ | 12 | (1.0) |
| Transdermal antipsychotics | 2 | (0.2) |
| Any transdermal atypical antipsychotics | 2 | (0.2) |
| SDA | 2 | (0.2) |
| Blonanserin | 2 | (0.2) |
| Injectable antipsychotics | 115 | (9.5) |
| Injectable typical antipsychotics | 109 | (9.0) |
| Injectable atypical antipsychotics | 11 | (0.9) |
| Benzamides | 2 | (0.2) |
| Sulpiride | 2 | (0.2) |
| Butyrophenones | 56 | (4.6) |
| Haloperidol | 55 | (4.6) |
| Haloperidol (LAI) | 2 | (0.2) |
| Timiperone | 0 | (0.0) |
| Phenothiazines | 55 | (4.6) |
| Chlorpromazine | 42 | (3.5) |
| Fluphenazine (LAI) | 1 | (0.1) |
| Levomepromazine | 9 | (0.7) |
| Perphenazine | 4 | (0.3) |
| DPA | 5 | (0.4) |
| Aripiprazole (LAI) | 5 | (0.4) |
| MARTA | 5 | (0.4) |
| Olanzapine | 5 | (0.4) |
| SDA | 3 | (0.2) |
| Paliperidone (LAI) | 2 | (0.2) |
| Risperidone (LAI) | 1 | (0.1) |

Abbreviations: DPA, Dopamine partial agonist; LAI, Long acting injection; MARTA, Multi-acting receptor targeted antipsychotic; SDA, Serotonin-dopamine antagonist.

^†^Zotepine is counted as a typical antipsychotic drug in the Drug class and as an SDA in the Drug subclass.

# **Table S3.** Typical or atypical antipsychotics prescribed 180 days before and during pregnancy and 180 days after delivery, excluding injectable prochlorperazine (N=120,735).

|  | Before pregnancy | | | | | | | |  | During pregnancy | | | | | | | | | | |  | Postpartum | | | | | | | |
| --- | --- | --- | --- | --- | --- | --- | --- | --- | --- | --- | --- | --- | --- | --- | --- | --- | --- | --- | --- | --- | --- | --- | --- | --- | --- | --- | --- | --- | --- |
|  | Within  -180 days | |  | -180 to  -91 days | |  | -90 to  -1 days | |  | Any  trimester | |  | First  trimester | |  | Second  trimester | |  | Third  trimester^†^ | |  | Within  180 days | |  | 1 to  90 days | |  | 91 to  180 days | |
|  | n | (Per 10,000) |  | n | (Per 10,000) |  | n | (Per 10,000) |  | n | (Per 10,000) |  | n | (Per 10,000) |  | n | (Per 10,000) |  | n | (Per 10,000) |  | n | (Per 10,000) |  | n | (Per 10,000) |  | n | (Per 10,000) |
| Any antipsychotics | 967 | (80.1) |  | 818 | (67.8) |  | 795 | (65.8) |  | 809 | (67.0) |  | 656 | (54.3) |  | 415 | (34.4) |  | 467 | (38.8) |  | 1023 | (84.7) |  | 820 | (67.9) |  | 714 | (59.1) |
| Any typical antipsychotics | 363 | (30.1) |  | 273 | (22.6) |  | 250 | (20.7) |  | 243 | (20.1) |  | 143 | (11.8) |  | 71 | (5.9) |  | 118 | (9.8) |  | 451 | (37.4) |  | 349 | (28.9) |  | 227 | (18.8) |
| Any atypical antipsychotics | 696 | (57.6) |  | 615 | (50.9) |  | 607 | (50.3) |  | 633 | (52.4) |  | 552 | (45.7) |  | 376 | (31.1) |  | 374 | (31.1) |  | 680 | (56.3) |  | 540 | (44.7) |  | 560 | (46.4) |

^†^Denominator of percentages is the number of pregnant women who reached third trimester (N = 120,394).

# **Table S4.** The transdermal antipsychotics prescribed 180 days before and during pregnancy and 180 days after delivery (N=120,735).

|  | Before pregnancy | | | | | | | |  | During pregnancy | | | | | | | | | | |  | Postpartum | | | | | | | |
| --- | --- | --- | --- | --- | --- | --- | --- | --- | --- | --- | --- | --- | --- | --- | --- | --- | --- | --- | --- | --- | --- | --- | --- | --- | --- | --- | --- | --- | --- |
|  | Within  -180 days | |  | -180 to  -91 days | |  | -90 to  -1 days | |  | Any  trimester | |  | First  trimester | |  | Second  trimester | |  | Third  trimester^†^ | |  | Within  180 days | |  | 1 to  90 days | |  | 91 to  180 days | |
|  | n | (Per 10,000) |  | n | (Per 10,000) |  | n | (Per 10,000) |  | n | (Per 10,000) |  | n | (Per 10,000) |  | n | (Per 10,000) |  | n | (Per 10,000) |  | n | (Per 10,000) |  | n | (Per 10,000) |  | n | (Per 10,000) |
| Any transdermal antipsychotics | 0 | (0.0) |  | 0 | (0.0) |  | 0 | (0.0) |  | 1 | (0.1) |  | 0 | (0.0) |  | 1 | (0.1) |  | 1 | (0.1) |  | 2 | (0.2) |  | 2 | (0.2) |  | 1 | (0.1) |
| Any transdermal atypical antipsychotics | 0 | (0.0) |  | 0 | (0.0) |  | 0 | (0.0) |  | 1 | (0.1) |  | 0 | (0.0) |  | 1 | (0.1) |  | 1 | (0.1) |  | 2 | (0.2) |  | 2 | (0.2) |  | 1 | (0.1) |
| SDA | 0 | (0.0) |  | 0 | (0.0) |  | 0 | (0.0) |  | 1 | (0.1) |  | 0 | (0.0) |  | 1 | (0.1) |  | 1 | (0.1) |  | 2 | (0.2) |  | 2 | (0.2) |  | 1 | (0.1) |
| Blonanserin | 0 | (0.0) |  | 0 | (0.0) |  | 0 | (0.0) |  | 1 | (0.1) |  | 0 | (0.0) |  | 1 | (0.1) |  | 1 | (0.1) |  | 2 | (0.2) |  | 2 | (0.2) |  | 1 | (0.1) |

^†^Denominator of percentages is the number of pregnant women who reached third trimester (N = 120,394).

# **Table S5.** Antipsychotics newly prescribed from 90 days before pregnancy to 180 days postpartum, excluding injectable prochlorperazine (N=120,735).

|  | Before pregnancy | | | |  |  | During pregnancy | | | | | |  | Postpartum | | | |
| --- | --- | --- | --- | --- | --- | --- | --- | --- | --- | --- | --- | --- | --- | --- | --- | --- | --- |
|  | -180 to  -91 days | | -90 to  -1 days | |  |  | First  trimester | | Second  trimester | | Third  trimester^†^ | |  | 1 to  90 days | | 91 to  180 days | |
|  | n | (Per 10,000) | n | (Per 10,000) |  |  | n | (Per 10,000) | n | (Per 10,000) | n | (Per 10,000) |  | n | (Per 10,000) | n | (Per 10,000) |
| Any antipsychotics | 818 | (67.8) | 149 | (12.3) |  |  | 77 | (6.4) | 36 | (3.0) | 93 | (7.7) |  | 360 | (29.8) | 165 | (13.7) |
| Any typical antipsychotics | 273 | (22.6) | 82 | (6.8) |  |  | 40 | (3.3) | 13 | (1.1) | 69 | (5.7) |  | 246 | (20.4) | 79 | (6.5) |
| Any atypical antipsychotics | 615 | (50.9) | 73 | (6.0) |  |  | 37 | (3.1) | 24 | (2.0) | 24 | (2.0) |  | 132 | (10.9) | 96 | (8.0) |
| Any oral antipsychotics | 815 | (67.5) | 146 | (12.1) |  |  | 75 | (6.2) | 35 | (2.9) | 51 | (4.2) |  | 346 | (28.7) | 163 | (13.5) |
| Any oral typical antipsychotics | 269 | (22.3) | 79 | (6.5) |  |  | 38 | (3.1) | 12 | (1.0) | 27 | (2.2) |  | 228 | (18.9) | 76 | (6.3) |
| Any oral atypical antipsychotics | 613 | (50.8) | 73 | (6.0) |  |  | 37 | (3.1) | 24 | (2.0) | 24 | (2.0) |  | 132 | (10.9) | 96 | (8.0) |
| Benzamides | 147 | (12.2) | 60 | (5.0) |  |  | 25 | (2.1) | 8 | (0.7) | 21 | (1.7) |  | 200 | (16.6) | 54 | (4.5) |
| Nemonapride | 0 | (0.0) | 0 | (0.0) |  |  | 0 | (0.0) | 0 | (0.0) | 0 | (0.0) |  | 0 | (0.0) | 0 | (0.0) |
| Sultopride | 0 | (0.0) | 0 | (0.0) |  |  | 0 | (0.0) | 0 | (0.0) | 0 | (0.0) |  | 0 | (0.0) | 0 | (0.0) |
| Sulpiride | 145 | (12.0) | 60 | (5.0) |  |  | 25 | (2.1) | 7 | (0.6) | 21 | (1.7) |  | 200 | (16.6) | 54 | (4.5) |
| Tiapride | 2 | (0.2) | 0 | (0.0) |  |  | 0 | (0.0) | 1 | (0.1) | 0 | (0.0) |  | 0 | (0.0) | 0 | (0.0) |
| Butyrophenones | 24 | (2.0) | 1 | (0.1) |  |  | 0 | (0.0) | 1 | (0.1) | 0 | (0.0) |  | 3 | (0.2) | 1 | (0.1) |
| Bromperidol | 5 | (0.4) | 0 | (0.0) |  |  | 0 | (0.0) | 0 | (0.0) | 0 | (0.0) |  | 0 | (0.0) | 0 | (0.0) |
| Haloperidol | 19 | (1.6) | 1 | (0.1) |  |  | 0 | (0.0) | 1 | (0.1) | 0 | (0.0) |  | 3 | (0.2) | 1 | (0.1) |
| Pimozide | 0 | (0.0) | 0 | (0.0) |  |  | 0 | (0.0) | 0 | (0.0) | 0 | (0.0) |  | 0 | (0.0) | 0 | (0.0) |
| Pipamperone | 0 | (0.0) | 0 | (0.0) |  |  | 0 | (0.0) | 0 | (0.0) | 0 | (0.0) |  | 0 | (0.0) | 0 | (0.0) |
| Spiperone | 0 | (0.0) | 0 | (0.0) |  |  | 0 | (0.0) | 0 | (0.0) | 0 | (0.0) |  | 0 | (0.0) | 0 | (0.0) |
| Timiperone | 0 | (0.0) | 0 | (0.0) |  |  | 0 | (0.0) | 0 | (0.0) | 0 | (0.0) |  | 0 | (0.0) | 0 | (0.0) |
| Iminodibenzyl | 0 | (0.0) | 0 | (0.0) |  |  | 0 | (0.0) | 0 | (0.0) | 0 | (0.0) |  | 0 | (0.0) | 0 | (0.0) |
| Clocapramine | 0 | (0.0) | 0 | (0.0) |  |  | 0 | (0.0) | 0 | (0.0) | 0 | (0.0) |  | 0 | (0.0) | 0 | (0.0) |
| Mosapramine | 0 | (0.0) | 0 | (0.0) |  |  | 0 | (0.0) | 0 | (0.0) | 0 | (0.0) |  | 0 | (0.0) | 0 | (0.0) |
| Indoles | 0 | (0.0) | 0 | (0.0) |  |  | 0 | (0.0) | 0 | (0.0) | 0 | (0.0) |  | 0 | (0.0) | 0 | (0.0) |
| Oxypertin | 0 | (0.0) | 0 | (0.0) |  |  | 0 | (0.0) | 0 | (0.0) | 0 | (0.0) |  | 0 | (0.0) | 0 | (0.0) |
| Phenothiazines | 103 | (8.5) | 18 | (1.5) |  |  | 13 | (1.1) | 3 | (0.2) | 6 | (0.5) |  | 26 | (2.2) | 21 | (1.7) |
| Carpipramine | 0 | (0.0) | 0 | (0.0) |  |  | 0 | (0.0) | 0 | (0.0) | 0 | (0.0) |  | 0 | (0.0) | 0 | (0.0) |
| Chlorpromazine | 43 | (3.6) | 4 | (0.3) |  |  | 3 | (0.2) | 0 | (0.0) | 0 | (0.0) |  | 8 | (0.7) | 5 | (0.4) |
| Fluphenazine | 3 | (0.2) | 0 | (0.0) |  |  | 0 | (0.0) | 0 | (0.0) | 0 | (0.0) |  | 0 | (0.0) | 0 | (0.0) |
| Levomepromazine | 44 | (3.6) | 4 | (0.3) |  |  | 0 | (0.0) | 1 | (0.1) | 1 | (0.1) |  | 7 | (0.6) | 4 | (0.3) |
| Perphenazine | 12 | (1.0) | 1 | (0.1) |  |  | 0 | (0.0) | 0 | (0.0) | 0 | (0.0) |  | 0 | (0.0) | 3 | (0.2) |
| Prochlorperazine | 7 | (0.6) | 10 | (0.8) |  |  | 10 | (0.8) | 2 | (0.2) | 5 | (0.4) |  | 12 | (1.0) | 9 | (0.7) |
| Propericiazine | 0 | (0.0) | 0 | (0.0) |  |  | 0 | (0.0) | 0 | (0.0) | 0 | (0.0) |  | 0 | (0.0) | 1 | (0.1) |
| Thioridazine | 0 | (0.0) | 0 | (0.0) |  |  | 0 | (0.0) | 0 | (0.0) | 0 | (0.0) |  | 0 | (0.0) | 0 | (0.0) |
| DPA | 305 | (25.3) | 36 | (3.0) |  |  | 10 | (0.8) | 5 | (0.4) | 2 | (0.2) |  | 31 | (2.6) | 34 | (2.8) |
| Aripiprazole | 295 | (24.4) | 34 | (2.8) |  |  | 9 | (0.7) | 5 | (0.4) | 2 | (0.2) |  | 29 | (2.4) | 30 | (2.5) |
| Brexpiprazole | 15 | (1.2) | 2 | (0.2) |  |  | 1 | (0.1) | 0 | (0.0) | 0 | (0.0) |  | 2 | (0.2) | 5 | (0.4) |
| MARTA | 262 | (21.7) | 26 | (2.2) |  |  | 17 | (1.4) | 17 | (1.4) | 15 | (1.2) |  | 82 | (6.8) | 54 | (4.5) |
| Asenapine | 7 | (0.6) | 0 | (0.0) |  |  | 0 | (0.0) | 0 | (0.0) | 0 | (0.0) |  | 2 | (0.2) | 1 | (0.1) |
| Clozapine | 0 | (0.0) | 0 | (0.0) |  |  | 0 | (0.0) | 0 | (0.0) | 0 | (0.0) |  | 0 | (0.0) | 0 | (0.0) |
| Olanzapine | 121 | (10.0) | 13 | (1.1) |  |  | 7 | (0.6) | 13 | (1.1) | 1 | (0.1) |  | 49 | (4.1) | 31 | (2.6) |
| Quetiapine | 147 | (12.2) | 15 | (1.2) |  |  | 10 | (0.8) | 5 | (0.4) | 14 | (1.2) |  | 36 | (3.0) | 24 | (2.0) |
| SDA | 171 | (14.2) | 17 | (1.4) |  |  | 10 | (0.8) | 7 | (0.6) | 10 | (0.8) |  | 41 | (3.4) | 28 | (2.3) |
| Blonanserin | 28 | (2.3) | 1 | (0.1) |  |  | 1 | (0.1) | 1 | (0.1) | 0 | (0.0) |  | 5 | (0.4) | 2 | (0.2) |
| Lurasidone | 0 | (0.0) | 1 | (0.1) |  |  | 0 | (0.0) | 0 | (0.0) | 0 | (0.0) |  | 0 | (0.0) | 3 | (0.2) |
| Paliperidone | 12 | (1.0) | 0 | (0.0) |  |  | 0 | (0.0) | 0 | (0.0) | 0 | (0.0) |  | 1 | (0.1) | 0 | (0.0) |
| Perospirone | 22 | (1.8) | 3 | (0.2) |  |  | 1 | (0.1) | 0 | (0.0) | 0 | (0.0) |  | 2 | (0.2) | 4 | (0.3) |
| Risperidone | 112 | (9.3) | 12 | (1.0) |  |  | 9 | (0.7) | 6 | (0.5) | 10 | (0.8) |  | 36 | (3.0) | 20 | (1.7) |
| Zotepine^‡^ | 7 | (0.6) | 0 | (0.0) |  |  | 0 | (0.0) | 0 | (0.0) | 0 | (0.0) |  | 0 | (0.0) | 0 | (0.0) |
| Any transdermal antipsychotics | 0 | (0.0) | 0 | (0.0) |  |  | 0 | (0.0) | 0 | (0.0) | 0 | (0.0) |  | 0 | (0.0) | 0 | (0.0) |
| Any transdermal atypical antipsychotics | 0 | (0.0) | 0 | (0.0) |  |  | 0 | (0.0) | 0 | (0.0) | 0 | (0.0) |  | 0 | (0.0) | 0 | (0.0) |
| SDA | 0 | (0.0) | 0 | (0.0) |  |  | 0 | (0.0) | 0 | (0.0) | 0 | (0.0) |  | 0 | (0.0) | 0 | (0.0) |
| Blonanserin | 0 | (0.0) | 0 | (0.0) |  |  | 0 | (0.0) | 0 | (0.0) | 0 | (0.0) |  | 0 | (0.0) | 0 | (0.0) |
| Any injectable antipsychotics | 11 | (0.9) | 5 | (0.4) |  |  | 2 | (0.2) | 1 | (0.1) | 42 | (3.5) |  | 24 | (2.0) | 4 | (0.3) |
| Any injectable typical antipsychotics | 6 | (0.5) | 4 | (0.3) |  |  | 2 | (0.2) | 1 | (0.1) | 42 | (3.5) |  | 24 | (2.0) | 3 | (0.2) |
| Any injectable atypical antipsychotics | 6 | (0.5) | 1 | (0.1) |  |  | 0 | (0.0) | 0 | (0.0) | 0 | (0.0) |  | 0 | (0.0) | 1 | (0.1) |
| Benzamides | 1 | (0.1) | 0 | (0.0) |  |  | 0 | (0.0) | 0 | (0.0) | 0 | (0.0) |  | 0 | (0.0) | 0 | (0.0) |
| Sulpiride | 1 | (0.1) | 0 | (0.0) |  |  | 0 | (0.0) | 0 | (0.0) | 0 | (0.0) |  | 0 | (0.0) | 0 | (0.0) |
| Butyrophenones | 5 | (0.4) | 2 | (0.2) |  |  | 2 | (0.2) | 0 | (0.0) | 5 | (0.4) |  | 17 | (1.4) | 3 | (0.2) |
| Haloperidol | 5 | (0.4) | 2 | (0.2) |  |  | 2 | (0.2) | 0 | (0.0) | 5 | (0.4) |  | 17 | (1.4) | 3 | (0.2) |
| Haloperidol (LAI) | 0 | (0.0) | 0 | (0.0) |  |  | 0 | (0.0) | 0 | (0.0) | 0 | (0.0) |  | 0 | (0.0) | 0 | (0.0) |
| Timiperone | 0 | (0.0) | 0 | (0.0) |  |  | 0 | (0.0) | 0 | (0.0) | 0 | (0.0) |  | 0 | (0.0) | 0 | (0.0) |
| Phenothiazines | 0 | (0.0) | 2 | (0.2) |  |  | 0 | (0.0) | 1 | (0.1) | 37 | (3.1) |  | 8 | (0.7) | 0 | (0.0) |
| Chlorpromazine | 0 | (0.0) | 0 | (0.0) |  |  | 0 | (0.0) | 1 | (0.1) | 36 | (3.0) |  | 4 | (0.3) | 0 | (0.0) |
| Fluphenazine (LAI) | 0 | (0.0) | 0 | (0.0) |  |  | 0 | (0.0) | 0 | (0.0) | 0 | (0.0) |  | 0 | (0.0) | 0 | (0.0) |
| Levomepromazine | 0 | (0.0) | 0 | (0.0) |  |  | 0 | (0.0) | 0 | (0.0) | 0 | (0.0) |  | 3 | (0.2) | 0 | (0.0) |
| Perphenazine | 0 | (0.0) | 2 | (0.2) |  |  | 0 | (0.0) | 0 | (0.0) | 1 | (0.1) |  | 1 | (0.1) | 0 | (0.0) |
| DPA | 3 | (0.2) | 1 | (0.1) |  |  | 0 | (0.0) | 0 | (0.0) | 0 | (0.0) |  | 0 | (0.0) | 0 | (0.0) |
| Aripiprazole (LAI) | 3 | (0.2) | 1 | (0.1) |  |  | 0 | (0.0) | 0 | (0.0) | 0 | (0.0) |  | 0 | (0.0) | 0 | (0.0) |
| MARTA | 1 | (0.1) | 0 | (0.0) |  |  | 0 | (0.0) | 0 | (0.0) | 0 | (0.0) |  | 0 | (0.0) | 1 | (0.1) |
| Olanzapine | 1 | (0.1) | 0 | (0.0) |  |  | 0 | (0.0) | 0 | (0.0) | 0 | (0.0) |  | 0 | (0.0) | 1 | (0.1) |
| SDA | 2 | (0.2) | 0 | (0.0) |  |  | 0 | (0.0) | 0 | (0.0) | 0 | (0.0) |  | 0 | (0.0) | 0 | (0.0) |
| Paliperidone (LAI) | 1 | (0.1) | 0 | (0.0) |  |  | 0 | (0.0) | 0 | (0.0) | 0 | (0.0) |  | 0 | (0.0) | 0 | (0.0) |
| Risperidone (LAI) | 1 | (0.1) | 0 | (0.0) |  |  | 0 | (0.0) | 0 | (0.0) | 0 | (0.0) |  | 0 | (0.0) | 0 | (0.0) |

Abbreviations: DPA, Dopamine partial agonist; LAI, Long acting injection; MARTA, Multi-acting receptor targeted antipsychotic; SDA, Serotonin-dopamine antagonist.

^†^Denominator of percentages is the number of pregnant women who reached third trimester (N= 120,394).

^‡^Zotepine is counted as a typical antipsychotic drug in the Drug class and as an SDA in the Drug subclass.

# **Estimation of pregnancy onset and delivery**

Abbreviations: GA, Gestational age.

Figure S1. Schematic illustration of the estimation of the dates of pregnancy onset and delivery based on the algorithm.

The date of pregnancy onset was estimated by subtracting the gestational age (GA) recorded as part of the diagnosis at a specific visit from the date of diagnosis. For example, when “delivery at week 39” was entered, 39 weeks and 0 days were subtracted (Figure S1). If a woman made several visits with diagnoses specifying the GA, the longest GA was applied, given that the GA at delivery represents the best obstetric estimate for clinical care ^1,2^; thus, the GA likely has greater accuracy in later stages of pregnancy. Based on an assessment using administrative data from a university hospital, 92.8% of the estimated pregnancy onset dates were within ±7 days of the gold standard date of pregnancy onset ^3^.

The delivery date was estimated based on delivery-related entries described in our previous reports and the birth months of infants ^3–5^. An algorithm was developed wherein the earliest dates of the selected diagnoses and surgical procedures were regarded as the delivery dates. In patients without such diagnoses or procedures, the earliest dates of any other delivery-related entries, including diagnoses, surgical or medical procedures, or injectable medications, were considered delivery dates. Based on an assessment using administrative data from a university hospital, 96.4% of the estimated delivery dates were within ±7 days of the gold standard delivery date ^3^. Given that birth months and years were available, delivery-related entries were used when the date was within the infant’s birth month. Delivery is not always covered by health insurance in Japan, and related information is not recorded when a delivery does not require procedures or medications that are covered by health insurance. In such patients, the 15th day of the neonatal birth month was considered the delivery date (Figure S1).

Labor induction is recommended in post-term pregnancies (pregnancies that reach or extend beyond 294 days of gestation) because of the increased risk of perinatal mortality ^2^. Thus, when the difference between the estimated dates of pregnancy onset and delivery exceeded 294 days, a gestational period of 294 days was uniformly assigned, and pregnancy onset was considered 294 days before the estimated delivery date. Pregnancies were divided into first (pregnancy onset to week 13 day 6 of gestation), second (week 14 day 0 to week 27 day 6), and third (week 28 day 0 to delivery) trimesters ^2^.

Reference:

1. Committee on Obstetric Practice, the American Institute of Ultrasound in Medicine, Society for Maternal-Fetal Medicine. Committee Opinion No 700: Methods for estimating the due date. *Obstet Gynecol.* 2017;129(5):e150-e154. <https://doi.org/10.1097/aog.0000000000002046>
2. Japan Society of Obstetrics and Gynecology and Japan Association of Obstetricians and Gynecologists. Guidelines for obstetrical practice in Japan 2020 edition. Tokyo: Japan Society of Obstetrics and Gynecology; 2020 (in Japanese).
3. Ishikawa T, Obara T, Nishigori H et al. Development of algorithms to determine the onset of pregnancy and delivery date using health care administrative data in a university hospital in Japan. *Pharmacoepidemiol Drug Saf.* 2018;27(7):751-762. <https://doi.org/10.1002/pds.4444>
4. Ishikawa T, Obara T, Nishigori H et al. Antihypertensives prescribed for pregnant women in Japan: Prevalence and timing determined from a database of health insurance claims. *Pharmacoepidemiol Drug Saf.* 2018;27(12):1325-1334. <https://doi.org/10.1002/pds.4654>
5. Ishikawa T, Obara T, Jin K et al. Examination of the prescription of antiepileptic drugs to prenatal and postpartum women in Japan from a health administrative database. *Pharmacoepidemiol Drug Saf.* 2019;28(6):804-811. <https://doi.org/10.1002/pds.4749>
